# A 2-transcript host cell signature distinguishes viral from bacterial diarrhea and it is influenced by the severity of symptoms

**DOI:** 10.1038/s41598-018-26239-1

**Published:** 2018-05-23

**Authors:** R. Barral-Arca, J. Pardo-Seco, F. Martinón-Torres, A. Salas

**Affiliations:** 1 Unidade de Xenética, Departamento de Anatomía Patolóxica e Ciencias Forenses, Instituto de Ciencias Forenses, Facultade de Medicina, Universidade de Santiago de Compostela, Galicia, Spain; 2 GenPoB Research Group, Instituto de Investigaciones Sanitarias (IDIS), Hospital Clínico Universitario de Santiago (SERGAS), Galicia, Spain; 3 Translational Pediatrics and Infectious Diseases, Hospital Clínico Universitario de Santiago, Santiago de Compostela, Spain; 4 GENVIP Research Group (www.genvip.org), Instituto de Investigación Sanitaria de Santiago (IDIS), Galicia, Spain

## Abstract

Recently, a biomarker signature consisting of 2-transcript host RNAs was proposed for discriminating bacterial from viral infections in febrile children. We evaluated the performance of this signature in a different disease scenario, namely a cohort of Mexican children (*n* = 174) suffering from acute diarrhea of different infectious etiologies. We first examined the admixed background of the patients, indicating that most of them have a predominantly Native American genetic ancestry with a variable amount of European background (ranging from 0% to 57%). The results confirm that the RNA test can discriminate between viral and bacterial causes of infection (t-test; *P*-value = 6.94×10^−11^; AUC = 80%; sensitivity: 68% [95% CI: 55%–79%]; specificity: 84% [95% CI: 78%–90%]), but the strength of the signal differs substantially depending on the causal pathogen, with the stronger signal being that of *Shigella* (*P*-value = 3.14 × 10^−12^; AUC = 89; sensitivity: 70% [95% CI: 57%–83%]; specificity: 100% [95% CI: 100%–100%]). The accuracy of this test improves significantly when excluding mild cases (*P*-value = 2.13 × 10^−6^; AUC = 85%; sensitivity: 79% [95% CI: 58%–95%]; specificity: 78% [95% CI: 65%–88%]). The results broaden the scope of previous studies by incorporating different pathogens, variable levels of disease severity, and different ancestral background of patients, and add confirmatory support to the clinical utility of these 2-transcript biomarkers.

## Introduction

There are increasing efforts aimed at developing host biomarkers allowing to distinguish viral from bacterial infections in febrile children^1^. The interest stems not only from the need to discriminate potentially life-threatening bacterial infections from viral infections, but also to avoid the unnecessary prescription of empirical antibiotic therapy irrespective of severity. The overuse of antibiotics worldwide is accelerating antimicrobial resistance (AMR), which is recognized as one of the greatest threats to human health worldwide^2^. Furthermore, AMR research now constitutes one of the main programs of the World Health Organization (WHO; http://www.who.int/antimicrobial-resistance/).

There is an increasing interest in discovering diagnostic biomarkers from whole blood gene expression able to distinguish viral from bacterial diseases showing similar initial clinical phenotypes^3^. Two promising approaches to this issue based on a RNA signature have been published recently. The study by Mahajan *et al*.^4^ detected a 66-transcript signature in blood that allowed to differentiate bacterial from viral infection in young febrile infants. Herberg *et al*.^5^ have recently identified a host whole blood RNA transcriptomic signature based on only two genes, *FAM89A* and *IFLI44L*, and these results were recently validated by Kaforou and colleagues^6^, again in neonates. The possibility of using only two biomarkers to accurately differentiate viral from bacterial infections makes this approach particularly attractive for the design of a test in a simple device for use in health institutions or as a point of care test^5^. However, despite of the encouraging results of these studies, further effort is required to evaluate the accuracy and clinical utility of these biomarkers in different clinical settings before they can be turned into a clinically applicable test.

At the same time, the implications of the ancestral background of patients in transcription patterns is very poorly approached in the literature. However, some studies demonstrated that gene expression can depend on the ancestry background owing to existing differential frequencies at regulatory polymorphisms in populations^7^. Taking the current evidence into account, we propose here a new approach that allows to infer the ancestry from transcriptomic data without the need for an *ad hoc* test. The inferred ancestry can then be used to evaluate the possible dependence of expression on genetic background.

The present study differs from previous efforts^5^ in that: (*i*) the performance of the 2-transcript host RNA signature for discriminating bacterial from viral infection is evaluated in a real-life scenario of children suffering from acute diarrhea, (*ii*) the accuracy of the 2-transcipt test is evaluated for acute diarrhea caused by different pathogens, (*iii*) the discriminatory capacity of this test is measured for different levels of disease severity, and (*iv*) the dataset employed is from Mexico, thus allowing to test the accuracy of these biomarkers in a non-European cohort.

## Material and Methods

### Samples and data

We retrieved from the GEO database^8^ an RNA expression dataset obtained from a cohort (*n* = 174) of Mexican children, aged less than 10 years old, suffering from acute diarrhea associated with a single viral or bacterial pathogen, and without systemic complications (GEO accession number: GSE69529).

### Ancestral analysis from RNA data

In order to investigate the ancestral background of the biological samples analyzed, RNA data were preliminary processed for the extraction of DNA variant information. We used Opossum to pre-process RNA-seq reads prior to variant calling^9^. Subsequently, Platypus software was used as variant caller tool^10^. A total of 68,327 SNPs could be initially retrieved from the RNA data.

Analysis of ancestral background of patients was carried out using standard procedures (e.g.^11,12^). Inferring ancestral characteristics of the donors requires the use of reference continental populations. Genome data from reference populations were retrieved from two SNP genome repositories. From The 1000 Genomes Project^13^ (http://www.internationalgenome.org), we used the European-CEU and the African Yoruban-YRI datasets. From Reich *et al*.^14^ we used different population samples (Aymara, Maya, Pima, Quechua, etc) that represent the Native American ancestry, as published previously^15^; only the Native American variation of these datasets was used for the analysis (masked data).

We then intersected the SNPs retrieved from the RNA data and the two reference DNA datasets, resulting in a final set of 416 SNPs. It has been shown that a few hundred SNPs can provide accurate population estimates of continental ancestry^16^.

Next, we computed identity-by-state (IBS) values from SNP data using PLINK^17^. With the aim of exploring for clusters of genetic variation in the population sets analyzed, a Multidimensional Scaling (MDS) plot was built using a matrix of pairwise individual IBS values. MDS was performed using the function *cmdscale* (library *stats*) from R (http://www.r-project.org). In addition, we obtained maximum likelihood estimations of individual ancestries from multi-locus SNP data using ADMIXTURE software^18^.

### RNA data processing

First of all, we performed a quality control of the raw data obtained from the Sequence Read Archive (SRA) using FastaQC (http://www.bioinformatics.babraham.ac.uk/projects/fastqc) to ensure that there were no problems or biases in our data which might affect the downstream analysis. Subsequently, we used MultiQC^19^ to aggregate the results from FastaQC across many samples into a single plot. We eliminated several samples from our analysis [CA234, CA273, CA251, CA287, CA352, CA450, CA68, 8CA864, CA236] because they had either an incorrect G-C content or many overrepresented sequences^20^. After the quality control of the raw sequencing data, we selected 175 whole blood samples including children infected by rotavirus (*n* = 53), enteroaggregative *Escherichia coli* (EAEC) (*n* = 18), enteropathogenic *Escherichia coli* (EPEC) (*n* = 10), diffuse-adhering *Escherichia coli* (DAEC) (*n* = 21), *Salmonella* (*n* = 36), and *Shigella* (*n* = 36). A group of healthy controls was also available in the GEO database, but it was processed only here for the aim of ancestry analysis (because the comparisons of virus *versus* bacterial transcription patterns does not need a group of controls^5^;).

The whole transcriptome reads were mapped against the version of the human genome provided by Ensembl (version GRCh38_r90/release 90) using the ultrafast universal RNA-seq aligner STAR^21^. The aligned reads were reported in BAM format. We also used STAR for counting the number of reads that map to each gene.

Using the Integrative Genomics Viewer (IGV)^22^ we visualized where the mapped reads were positioned in the genes of interest. Last, we normalized the reads using several methods, including RPKM (Reads per million mapped reads), TMM (Trimmed mean of M-values), CQN (Conditional quantile normalization), and Deseq2 implemented in the *Deseq2* package of the R statistical software (https://www.r-project.org). All the methods yielded virtually the same result; Deseq2 was finally chosen.

Boxplots were built using the R package *beeswarm* (https://cran.r-project.org/web/packages/beeswarm) to represent the Disease Risk Score (DRS)^5^ according to the type of infection and pathogen. Given that the 2-transcript signature in our dataset follows a normal distribution (Shapiro test; *P*-value > 0.05), we used the T-test for independent samples to evaluate the statistical significance of the differential expression in bacterial *vs*. viral infected patients. In addition, to evaluate the predictive accuracy of the 2-transcript signature we constructed Receiver Operating Characteristic (ROC) curves and the area under the curves (AUC) using the *pROC* package in R. The threshold value, defined as the point on the ROC curve that maximized sensitivity and specificity, was calculated using the R package *OptimalCutPoints*^23^. The calculation of the confidence intervals for sensitivity and the specificity was based on a stratified bootstrap resampling.

## Results

### Ancestry analysis

**Figure 1:**
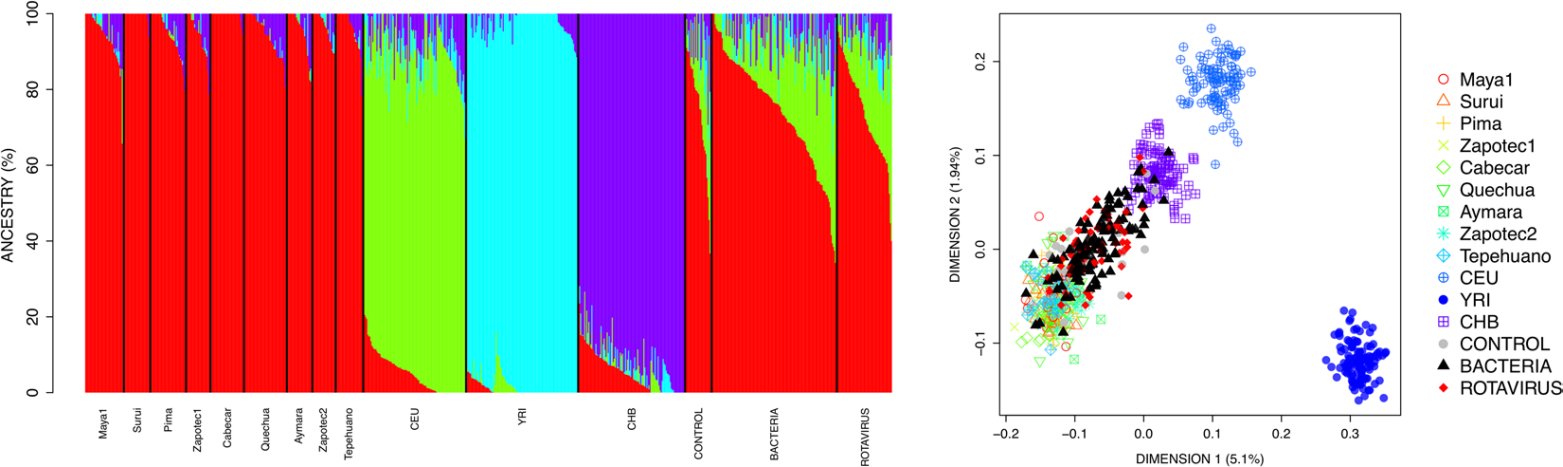
Ancestry analysis of RNA samples based on SNP data. (**A**) Barplot of maximum likelihood estimations of individual ancestries, indicating that most patients have a main Native American ancestry, while European ancestry is present in a few of them with values ranging from 0% to 57.7%. (**B**) MDS plot of pairwise individual IBS values showing that the genome profile of patients and controls (labeled in the figure as control, bacteria and rotavirus) falls close to the Native America cluster (identified by several population samples of Native American ethnic origin), and some of the samples slightly displaced towards the European pole represented by CEU in Dimensions 1 and 2.

Admixture analysis of the RNA data in virus, bacteria, and control cohorts indicates that the predominant ancestry is clearly Native American (average values: virus = 76.8% [range = 44.7-96.8], bacteria = 76.9% [range = 33.4-100], controls = 77.7 [range = 46–93.7]); the three datasets contain substantial levels of European ancestry too (average values: virus = 19.4%, bacteria = 19.4%; controls = 17.4%) (Fig. 1A). On average, Native American and European ancestries in patients are 77.0% and 19.2%, respectively.

The MDS plot of virus, bacteria, and control groups, together with the three main continental reference populations is in good concordance with the admixture analysis (Fig. 1B). There are three main poles of ancestry in the MDS plots, coinciding with the main three continental groups: Native Americans, Europeans, and Africans. Patients and controls fall closely related to the Native American cluster; a few samples are slightly projected the European cluster, indicating a more marked European ancestry in their genomes.

### The 2-transcript RNA signature in virus *versus* bacteria

**Figure 2:**
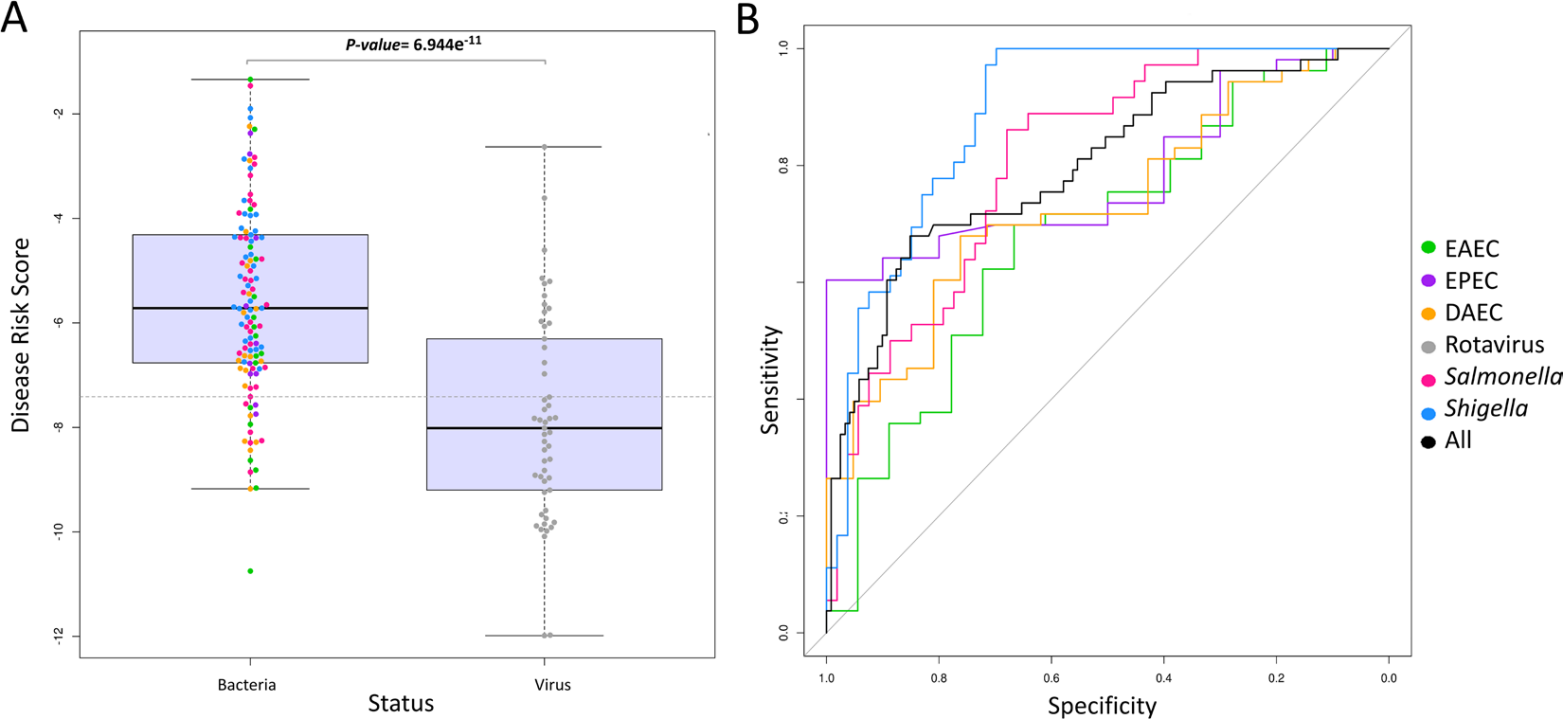
Classification performance based on the 2-transcript DRS combined as [log2(*FAM89*expresion)–log2(*ILF44L*expression)]. (**A**) Box and whisker plot of DRS: the horizontal lines in the boxes indicate the median of each group; the lower and upper edges of boxes reflect interquartile ranges, and the whiskers are <1 times the interquartile range; the horizontal grey line is the DRS threshold that maximizes the AUC when comparing patients with viral and bacterial infections. (**B**) ROC curves of different bacteria compared to rotavirus; the different colors identify the type of bacteria according to the inset legend. AUC values are provided in Table 1.

We first examined if patients clustered according to their disease status (bacterial *vs*. viral infection) when applying the DRS. We generated a one-dimensional scatter plot with closely-packed but non-overlapping points (Fig. 2A), which shows a statistically significant difference in the DRS of children affected by bacterial diarrhea compared to those suffering from viral diarrhea (*P*-value = 6.94 × 10^−11^). The results are consistent with those obtained by Herberg *et al*.^5^ in that a higher DRS indicates bacterial infection, whereas a lower DRS indicates viral infection.

The diagnostic accuracy of the test to discriminate viral from bacterial diarrhea was evaluated using ROC analysis (Fig. 2B), and considering different scenarios: (*i*) all the bacterial infections against rotavirus, and (*ii*) each bacterial pathogen (EAEC, DAEC, EPEC, *Salmonella*, and *Shigella*) against rotavirus. For the first scenario, the ROC curve indicates that the accuracy of the test is ~80%, a value that is comparable to that reported by Herberg *et al*. (namely, AUC = ~90%). At the same time, the diagnostic performance of the 2-transcript test seems to be pathogen-dependent (Fig. 2B), with some bacterial infections showing more distinctive pattern than viral infections; for example, the AUC for *Shigella* is very high (AUC = 89.0%; 95% CI: 82.5–95.6), while this is more moderate for EAEC (AUC = 68.7%; 95% CI: 54.0-83.3). In most of the comparisons carried out considering the different groups of bacteria independently, the differential expression in bacterial *vs*. viral infections was highly statistically significant, surpassing the Bonferroni threshold; in cases not showing statistical significance, this could be due to small sample sizes, since most are close to the limit of the significance threshold; Table 1).

**Table 1:** AUC values and DRS for different pathogens. Figures in round brackets show the CI 95% (2,000 bootstrap replicates).

	***n***	**AUC**	**Sensitivity**	**Specificity**	**DRS**	***P*** **-value**
***All patients***						
Rotavirus	53	—	—	—	—	
EAEC	18	68.7 (54.0-83.3)	69.8 (56.6–81.1)	66.7 (44.4–88.9)	−6.76	2.160 × 10^–2^
EPEC	10	78.6 (66.1–90.9)	60.4 (47.2–73.6)	90.0 (70.0–100.0)	−7.74	8.879 × 10^−3^
DAEC	21	73.6 (61.7–85.5)	67.9 (54.7–81.1)	76.2 (57.1–90.5)	−7.21	2.494 × 10^−3*^
*Salmonella*	36	80.8 (71.9–89.6)	67.9 (54.7–79.2)	83.3 (69.4–94.4)	−7.41	6.927 × 10^−7*^
*Shigella*	36	89.0 (82.5–95.6)	69.8 (56.6–83.0)	100.0 (100.0–100.0)	−6.88	3.140 × 10^−12*^
All Bacteria	121	80.0 (72.6–87.4)	67.9 (54.7–79.2)	84.3 (77.7–90.1)	−7.41	6.944 × 10^−11*^
***Severe & Moderate***						
Rotavirus	19	—	—	—	—	
EAEC	6	58.8 (28.4–89.2)	79.0 (57.9–94.7)	33.3 (0.0–66.7)	−6.25	6.102 × 10^−1^
EPEC	3	84.2 (52.3–100.0)	100.0 (100.0–100.0)	66.7 (0.0–100.0)	−4.37	1.780 × 10^−1^
DAEC	6	86.0 (66.6–100.0)	89.5 (73.7–100.0)	66.7 (33.3–100.0)	−5.80	4.057 × 10^−2^
*Salmonella*	12	83.3 (69.0–97.6)	68.4 (47.4–89.5)	83.3 (58.3–100.0)	−7.41	5.879 × 10^−3*^
*Shigella*	22	93.5 (86.6–100.0)	73.7 (52.6–94.7)	100.0 (100.0–100.0)	−6.75	2.219 × 10^−7*^
All bacteria	49	85.3 (75.7–94.9)	79.0 (57.9–94.7)	77.6 (65.3–87.8)	−6.40	2.126 × 10^−6*^
***Mild***						
Rotavirus	34	—	—	—	—	—
EAEC	12	73.5 (57.6–89.5)	67.7 (47.1–82.4)	75.0 (0.5–100.0)	−6.76	1.818 × 10^−2^
EPEC	7	74.8 (59.3–90.3)	67.7 (47.1–82.4)	100.0 (100.0–100.0)	−7.57	4.668 × 10^−2^
DAEC	15	69.0 (53.9–84.2)	64.7 (47.1–79.4)	80.0 (60.0–100.0)	−7.78	3.601 × 10^−2^
*Salmonella*	24	80.0 (68.6–91.4)	67.7 (53.0–82.4)	66.7 (83.3–95.8)	−7.55	4.698 × 10^−5*^
*Shigella*	14	84.2 (73.4–95.1)	67.7 (52.9–82.4)	100.0 (100.0–100.0)	−6.88	1.157 × 10^−5*^
All bacteria	72	77.0 (66.5–87.4)	67.7 (50.0–82.4)	86.1 (77.8–93.1)	−7.57	8.154 × 10^−6*^

*P*-values evaluate the significance of the difference between bacterial pathogen *vs*. viral infection under a t-test; an asterisk. (*) indicates those values surpassing a Bonferroni correction for multiple tests. Note that the reference viral pathogen in our study is rotavirus (for which there is only very little information available in the literature; e.g.^43^).

**Figure 3:**
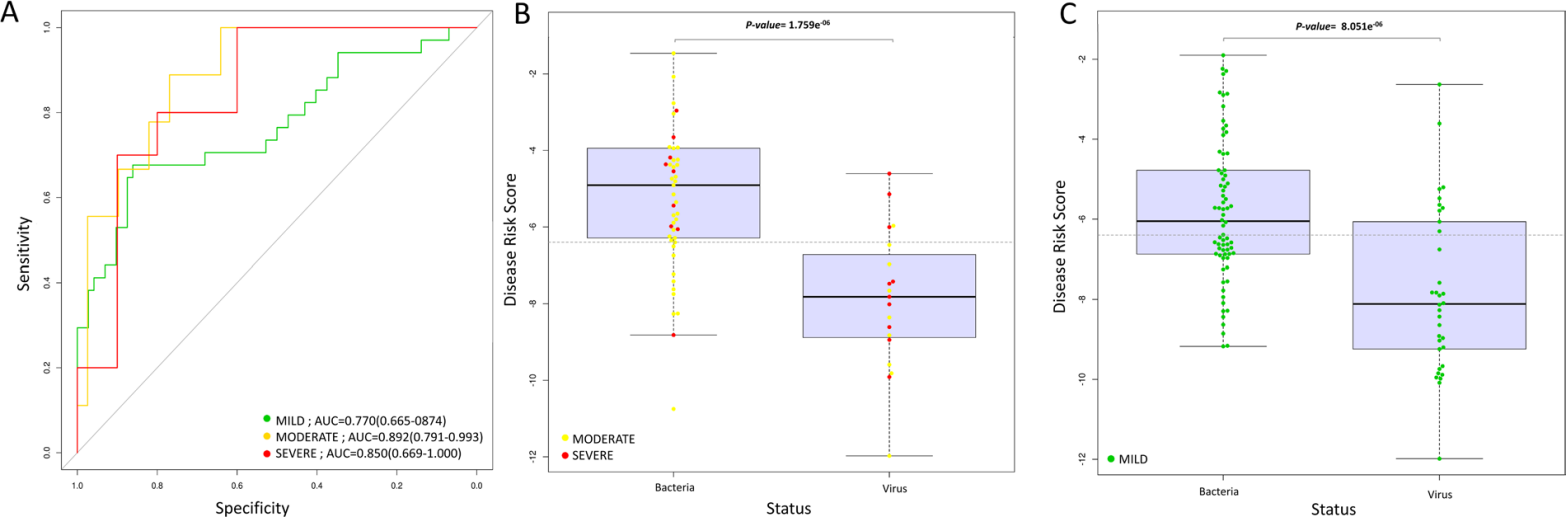
Classification performance based on the 2-transcript DRS combined as [log2(*FAM89*expresion)–log2(*ILF44L*expression)] and considering different levels of severity. (**A**) ROC curves of different bacteria compared to rotavirus; AUC values by pathogen are provided in Table 1; and (**B**) and (**C**) box and whisker plots of DRS for moderate plus severe cases and mild cases, respectively.

Most interesting is the observation that the level of severity affects the accuracy of the 2-biomarker test. Thus, patients with mild disease introduce noise in the capacity of the two biomarkers to characterize bacterial from viral infections; when these patients are excluded, the signal improves substantially as reflected in the boxplots in Fig. 3B,C; the difference with respect to viral infection is still statistically significant in both groups of patients, severe + moderate (*P*-value = 2.13 × 10^−06^) and mild (*P*-value = 8.15 × 10^−06^) cases (Table 1). This difference is also evident when examining the ROCs obtained by considering different levels of severity (Fig. 3A; AUC_MILD_ = 77.0% *vs*. AUC_SEVERE+MODERATE_ = 85.3%; AUC_SEVERE_ = 85.0%; AUC_MODERATE_ = 89.2%).

Finally, a correlation analysis between the 2-transcript test and the different ancestral coefficients indicate that expression of these two genes is not correlated with ancestral background (Fig. S1 from Supplementary Data).

## Discussion

It is common practice in hospitals to administer antibiotics to febrile children until the culture results are available^5^. Antibiotics are generally prescribed out of fear of missing a bacterial infection, and such practice is contributing to the rise of drug-resistant pathogens all over the world^1^. To give some examples, according to the data presented by Craig *et al*.^24^ on a prospective cohort study of >15,700 febrile illnesses, antibiotics were prescribed acutely in 66% to 81% of children (depending on the type of infection); while 20% of patients without bacterial infection received also antibiotic treatment. According to another study on meningitis, no pathogen was identified in 50%–80% of the patients but most of them received antibiotics, and almost 50% were (probably unnecessarily) hospitalized for more than a week^25^. Nijman *et al*.^26^ stated that “*a prediction model should discriminate between children at high and low risk of serious bacterial infections […] This is helpful to identify children who may need further tests, such as additional blood tests or chest radiography, and guide the need for antibiotic prescribing*”. On the other hand, culture of bacteria from normally sterile sites may be negative in scenarios where the infection resides in inaccessible sites, when the patient received antibiotic previously, or when the culture media selected fail to grow certain bacteria^5,27^. According to the meta-analysis carried out by Iroth Tam *et al*.^28^, blood culture identifies organisms in only a very small percentage of patients (~5%) suffering pediatric community-acquired pneumonia (CAP) (predominantly *S. pneumoniae*); this result was also corroborated by Kwon *et al*.^29^, who claimed the low utility of blood culture in CAP in an observational study considering >2,700 patients. In addition, while some authors defend the utility of MALDI-TOF as a new rapid technology for microbial identification^30^, the results from conventional cultures of bacteria may take several days^5,27,30^, so physicians commonly make the decision to administer preventive antibiotic treatment^25,31^.

In this scenario, developing a cost-effective device to differentiate viral from bacterial infections in early stages is one of the biggest challenges in medical research. In 2016, Herberg *et al*.^5^, published a study that shed new light on this problem as they identified a simple blood 2-transcript RNA expression signature that distinguishes viral from bacterial infection in febrile children.

Our study adds further support to these findings and also broadens the scope of the initial research. First, the Herberg *et al*.^5^ study focused on a number of well-selected viral and bacterial febrile patients that needed intensive care or had severe infections (e.g. sepsis); whereas the data set investigated here consisted of children affected by diarrhea caused by several pathogens, and thus a cohort that might represent a frequent scenario in emergency departments or outpatient clinics. Our results indicate that the 2-transcript test performs differently in different infectious scenarios, with the best results shown for diarrhea caused by *Shigella*.

Second, the majority of the pathogens included in the Mexican database (e.g. *Shigella, Rotavirus*) had not been tested in the original study or, as in the case of *E. coli and Salmonella*, our study adds more cases and different strains. This supports the conclusion that the test seems to perform differently depending on the pathogen.

Third, the database explored here included children with mild, moderate and severe symptomatology, which allowed us to assess the 2-transcript signature ranked by levels of severity. The results show that the test performs better in patients suffering from moderate to severe symptoms of diarrhea.

Fourth, we measured the potential dependence of the 2-transcript test on the ancestral background of patients. To the best of our knowledge, this is the first time that genetic background is inferred from transcriptomic data aiming at evaluating its potential effect on gene expression. Commonly, self-reported ancestry is used to stratify the statistical analyses; e.g.^32^; and in a number of studies, although well-differentiated ancestral groups were analyzed, there is no indication on how the ancestral background was assessed^33^. The exception to this common practice is Serrano-Gómez *et al*.^34^; this study used ancestry-informative markers (AIMs)^16^ to measure the ancestry as a potential modifier of gene expression in breast cancer. The original dataset used by Herberg *et al*.^5^ included patients of main European ancestry (UK, Spain, the Netherlands, and USA); while our sample represents a Mesoamerican population with predominantly Native American ancestry but variable levels of admixture. However, our results indicate that the 2-trancript test performs well in a different background to the one used for its discovery^35^, and that there is no correlation between the expression signal and the ancestral background of patients.

Last but not least, the original study used data provided by different microarray platforms; the batch effects were targeted computationally. The entire Mexican dataset was generated using RNA-seq and the sequencer Illumina HiSeq2500, and therefore batch effects should be minimal.

There are several further considerations from the present study. The results indicate that the 2-biomarkers test performs much better in severe patients (more closely related to those examined by Herberg *et al*.^5^). We have exclusively analyzed cases with a single infecting pathogen, but it would most interesting to further explore the behavior of this 2-biomarker test in scenarios of coinfection^36–38^. The translation of this 2-biomarker test into a clinical setup still needs further validation; and this validation would require the analysis of more virus and bacteria scenarios, as well as different time points in the course of the same infection, or the case of parasitic infections. Despite its limitations, the present study (together with the previous ones^5,6^) represents a step forward towards a bench to bedside test that could help make decisions for antibiotic administration^3^. It is now possible to test transcription signatures using portable devices^39–41^, so these techniques will be most helpful in small hospitals in rural and isolated areas, and in hospitals from developing countries. In addition, although the signal provided by *IFI44L* and *FAM89A* transcripts is not as strong in diarrhea patients as in more severe disease scenarios^5^, the evidence points to an important role of these two genes in the differential molecular mechanisms employed by virus and bacteria for infection. These observations should attract further research exploring the role of these genes and related pathways under the hypothesis that both could be key at understanding mechanisms of infections. To the best of our knowledge, there is very little information available for these two genes, but the *FAM89A* (interferon-induced protein 44-like) is known to exhibit antiviral activity^42^.

Summarizing, in all of the scenarios tested, our results suggest that these two biomarkers, *IFI44L* and *FAM89A* transcripts, provide a strong signal to differentiate bacterial from viral infections, a signal that is non-population dependent, and useful for discriminating a wide range of pathogens and different levels of severity. Further studies focusing on the time point of the disease, testing new (portable) devices for RNA typing, and targeting different populations worldwide and a wider spectrum of pathogens will be necessary to further confirm the accuracy of this biomarker test before it can be turned into a clinically applicable test.

## Electronic supplementary material

Supplementary Info
