# Supplementary material for: A 2-transcript host cell signature distinguishes viral from bacterial diarrhea and it is influenced by the severity of symptoms
Source: Sci Rep. 2018 May 23;8:8043. doi: 10.1038/s41598-018-26239-1 (PMC5966427; doi:10.1038/s41598-018-26239-1)

Supplementary Data

A 2-transcript host cell signature distinguishes viral from bacterial diarrhea and it is influenced by the severity of symptoms

Barral-Arca R, Pardo-Seco J, Martinón-Torres F, Salas A

Figure S1. Correlation analysis between ancestral coefficients obtained from admixture analysis and the signal provided by the 2-transcript test.

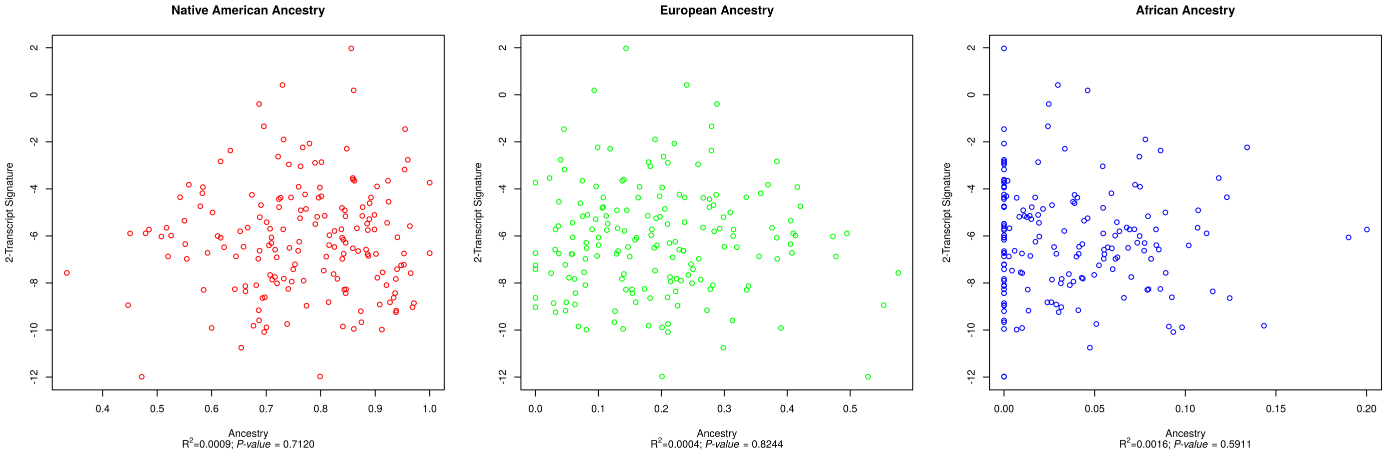

Supplement: Supplementary file 1 — Supplementary Info [file 41598_2018_26239_MOESM1_ESM.pdf]
